# Plexin A3 and Turnout Regulate Motor Axonal Branch Morphogenesis in Zebrafish

**DOI:** 10.1371/journal.pone.0054071

**Published:** 2013-01-21

**Authors:** Rajiv Sainath, Michael Granato

**Affiliations:** Department of Cell and Developmental Biology, University of Pennsylvania, Perelman School of Medicine, Philadelphia, Pennsylvania, United States of America; National Institutes of Health (NIH), United States of America

## Abstract

During embryogenesis motor axons navigate to their target muscles, where individual motor axons develop complex branch morphologies. The mechanisms that control axonal branching morphogenesis have been studied intensively, yet it still remains unclear when branches begin to form or how branch locations are determined. Live cell imaging of individual zebrafish motor axons reveals that the first axonal branches are generated at the ventral extent of the myotome via bifurcation of the growth cone. Subsequent branches are generated by collateral branching restricted to their synaptic target field along the distal portion of the axon. This precisely timed and spatially restricted branching process is disrupted in *turnout* mutants we identified in a forward genetic screen. Molecular genetic mapping positioned the *turnout* mutation within a 300 kb region encompassing eight annotated genes, however sequence analysis of all eight open reading frames failed to unambiguously identify the *turnout* mutation. Chimeric analysis and single cell labeling reveal that *turnout* function is required cell non-autonomously for intraspinal motor axon guidance and peripheral branch formation. *turnout* mutant motor axons form the first branch on time via growth cone bifurcation, but unlike wild-type they form collateral branches precociously, when the growth cone is still navigating towards the ventral myotome. These precocious collateral branches emerge along the proximal region of the axon shaft typically devoid of branches, and they develop into stable, permanent branches. Furthermore, we find that null mutants of the guidance receptor *plexin* A3 display identical motor axon branching defects, and time lapse analysis reveals that precocious branch formation in *turnout* and *plexin* A3 mutants is due to increased stability of otherwise short-lived axonal protrusions. Thus, *plexin A3* dependent intrinsic and *turnout* dependent extrinsic mechanisms suppress collateral branch morphogenesis by destabilizing membrane protrusions before the growth cone completes navigation into the synaptic target field.

## Introduction

During vertebrate development, motor axons navigate to their muscle targets where they generate elaborate axonal branches that synapse on multiple muscle fibers [1], [2]. In rodents, the number of axonal branches diminishes postnatally via synapse elimination to yield single innervation of adult muscle fibers [1], [2], while in lower vertebrates such as zebrafish the number of axonal branches remains high, resulting in poly innervation of adult muscle fibers [3]. The processes of motor axon guidance, neuromuscular synapse formation and even synapse elimination have been studied *in vivo* in great detail using genetic model systems [reviewed in 4,5,6]. In contrast the mechanisms that control motor axonal branching morphogenesis, including when branches begin to form and how branch locations are determined, has been studied mostly using cultured neurons and less in developing vertebrate embryos [7].

The stable, activity independent branching patterns formed during development are the product of branch formation, extension, stabilization and pruning. At the cellular level, branching begins with the localized accumulation of actin, and the de-bundling of microtubules at the nascent branch point, followed by the formation and extension of f-actin rich membrane protrusions into which microtubules subsequently extend [8], [9]. Both f-actin and microtubules are required for branch formation, and loss of either via administration of polymerization inhibiting drugs can suppress the formation of axon branches *in vitro* without changing axon length [8]. While microtubules stabilize axonal protrusions, several intracellular regulators have been implicated in the transition from axon protrusions into stable branches. These factors include the C. elegans ubiquitin ligase Rpm-1 [10]–[12], and the phosphatidylinositol 3-kinase (PI3K)/protein kinases AKT/glycogen synthase kinase 3 (GSK3) pathway in dorsal root ganglia neurons [10], [11].

The stabilization of protrusions into permanent axon branches produces one or more of three common patterns of axon branching. These common axon branching patterns are arborization, which occurs when multiple branches form in the target region at the axon terminal; bifurcation, the process of a growth cone splitting and forming two daughter branches, and collateral formation, which is the formation of branches along the axon shaft distally from the axon terminal [7]. Importantly, individual axons may exhibit a combination of these branching patterns, resulting in elaborate axonal branch morphologies characteristic for many neuronal cell types [13].

Many extracellular and intracellular molecules, including the axon guidance cues Netrin and Semaphorin 3A, the BDNF regulator Sprouty3, and the cell adhesion molecule cadherin 7 have been shown to influence motor axon branching [14]–[16]. For example, in mice, loss of the secreted inhibitory ligand *semaphorin 3A* results in a more branched dorsal root ganglion innervation [17]. While *semaphorin 3A* clearly plays a critical role in controlling axon branching, it has remained unclear *when* during development additional branches are made, whether they were permanent or pruned later, and if and how *semaphorin 3D* influences these processes. This is due in part due to the difficulties of live cell imaging during mammalian development, and in part due to the difficulties of labeling and imaging of identified, single neurons *in vivo*, essential to determine if the mutant phenotype is due to defects in frequency of generating protrusions, their extension or their stabilization into permanent branches.

In zebrafish, motor axonal branching patterns generated during embryonic development persist into adulthood, providing a unique system to analyze in live intact embryos the entire dynamic process by which nascent membrane protrusions develop into permanent axonal branches [3]. Here, we focus on the Caudal Primary (CaP) motor neuron, which is the first motor neuron whose axon exits from the spinal cord to pioneer into the ventral myotome, and the first motor neuron to form axonal branches [3]. Using live imaging in intact animals, we characterize the events that lead to an elaborate CaP axonal branching pattern, both via growth cone bifurcation and via collateral branch formation. In wild-type embryos, we uncover a precisely timed and spatially restricted branching process. In mutants of two genes, *turnout* and *plexin A3*, we identified in a forward genetic screen, this branching process is disrupted. Detailed analysis revealed a cell non-autonomous function for *turnout* in regulating motor guidance and axonal branching of all primary motor neurons. We find that *turnout* is dispensable for branches formed via growth cone bifurcation, and dispensable for total branch number, but that *turnout* controls when and where collateral branches form. Similarly, we show that the cell-autonomous Plexin A3 receptor controls the onset of CaP primary axon collateral branching as well as the location of the branches. Finally, we demonstrate that both *turnout* and *plexin A3* play critical role for collateral branching by regulating the stability of axonal protrusions.

## Results

### *turnout* is a Novel Mutant that Affects Motor Axon Development

**Figure 1:**
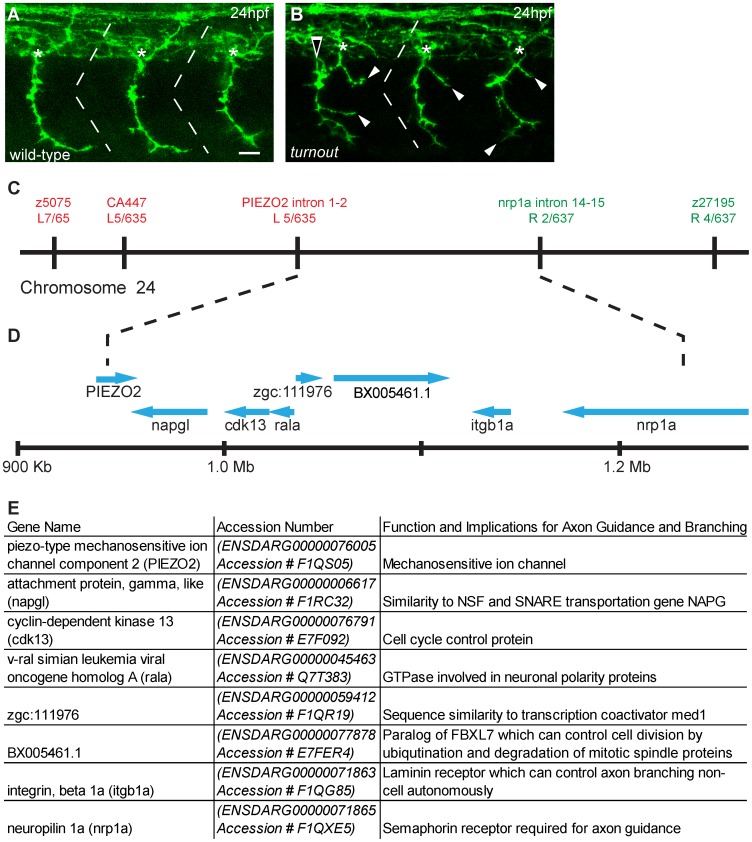
*turnout* mutants display guidance and branching defects and maps to chromosome 24 (A) Antibody staining of 24 hpf wild-type embryos to visualize motor axon projections. Note that axons exit the spinal cord at the midsegmental exit point (asterisk) with no branches. (B) In *turnout* mutants, motor axons exit the spinal cord at ectopic locations (open arrow), and also display long peripheral branches (arrowhead). (C) Genetic map surrounding the *turnout* locus. Genetic markers and the number of associated recombinant meioses to the left (red text) and to the right (green text) of the *turnout* mutation are indicated. (D) The physical map of the critical region and annotated genes in this interval. (E) List of annotated genes. Scale bar, 15 µm.

In an ongoing genetic screen for recessive mutations affecting motor axon guidance and peripheral branching [18], we identified a new mutant, *p5THBD,* which displays severe motor axonal defects. To quantify motor axon defects, we first used an anti-synaptotagmin antibody to visualize motor axon trajectories (also known as znp-1) [19]. In 24 hours post fertilization (hpf) wild-type embryos the pioneering primary motor axons have exited the spinal cord through the ventral root located at the center of each spinal hemisegment. From there they extend into the dorsal and ventral myotome, respectively (Fig. 1A) [3]. In contrast, *p5THBD* mutant embryos display two prominent motor axonal defects: exit from the spinal cord at ectopic positions (14% of hemisegments, n = 1208 hemisegments), and formation of peripheral branches (42%, n = 1208 hemisgements, Fig. 1B), which are never observed in 24 hpf wild-type embryos. Analysis of *p5THBD* embryos using various markers indicative of motor neuron specification (*islet1*, *islet2;* data not shown), myotome development (*slow myosin heavy chain*), somite polarity (*engrailed*), as well as for extracellular matrix components (*CSPG*), revealed no significant defects in any of these structures when compared to wild-type embryos (Fig. S1). Moreover, at 120 hpf *p5THBD* mutants inflate their swim bladder and are indistinguishable from their wild-type siblings. However, *p5THBD* mutants fail to survive to adulthood, suggesting that *p5THBD* function is required for additional yet unknown postembryonic processes.

To determine if the *p5THBD* phenotype is caused by mutations in genes known to regulate axonal branching, we mapped the mutation. Standard bulk segregate analysis revealed that the *p5THBD* mutation maps to chromosome 24 between the simple sequence length polymorphism markers z5075 and z27195 (Fig. 1C). Recombination mapping using additional molecular markers narrowed the genomic interval of the *p5THBD* mutation to a ∼300 Kb region between intronic makers of the *PIEZO2* and the *nrp1a* genes (Fig. 1C). This region contains eight annotated open reading frames, including exons 15–17, corresponding to amino acids 778–923, of the *plexin* co-receptor *nrp1a*, and the entire open reading frame of the *integrin beta 1a* gene (Fig. 1D). Sequence analysis revealed no missense or non-sense mutations in the open reading frame of the *integrin beta 1a* or the *plexin* co-receptor *nrp1a*. Moreover, *p5THBD* acts cell autonomously (see below), which is inconsistent with *nrp1a* being the affected gene. Sequence analysis of the open reading frames of the other six genes did reveal missense but no nonsense mutations. Mutants for any of these six genes have not been reported, and therefore we were unable to confirm or exclude if *p5THBD* is due to a mutation in any of these genes. Complete sequencing of the 300 kb interval will be required to definitively identify the molecular nature of the *turnout* gene. Since mutations in none of these eight genes have been reported in zebrafish, we named this mutant *turnout^p5THBD^*.

### *turnout* Mutants Affect Intraspinal Motor Axon Guidance and Peripheral Branch Formation

**Figure 2:**
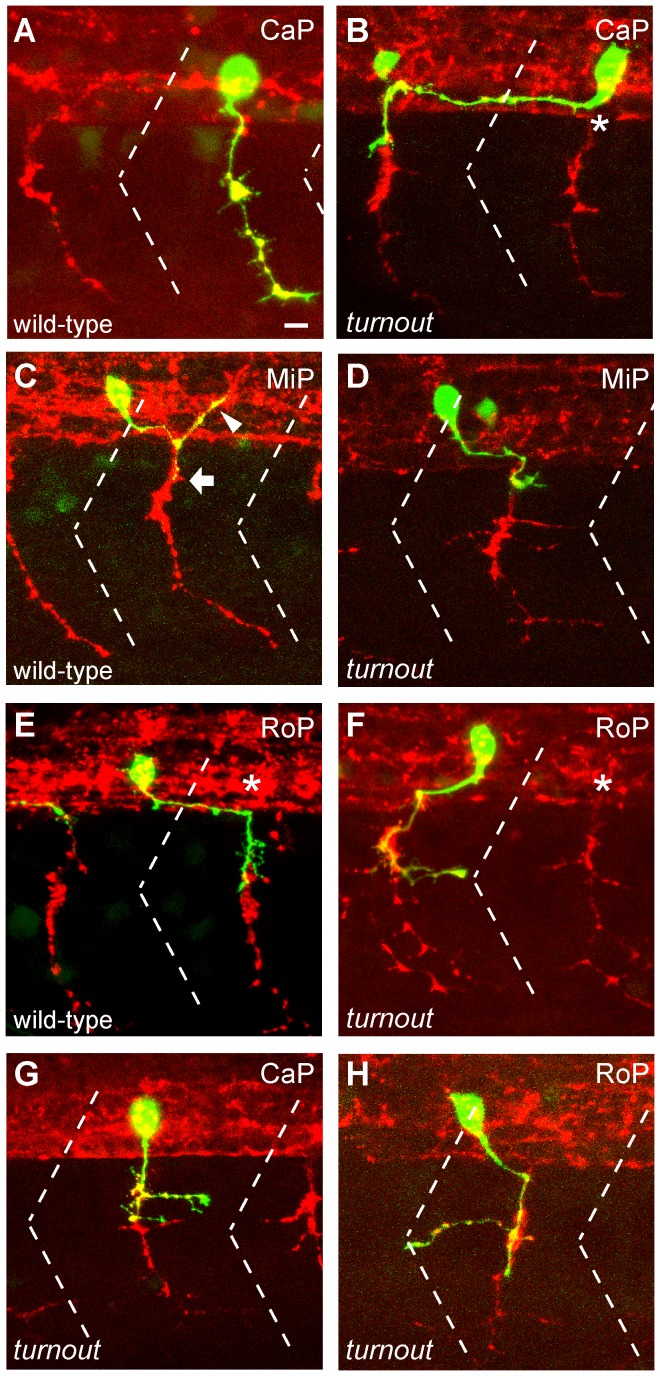
*turnout* controls guidance and branching of primary motor axons. (A, C, E) 24 hpf wild-type embryos injected with *mnx1:GFP* to visualize individual CaP, MiP and RoP motor neurons, and counter-stained with the SV2 antibody (red) to visualize the segmental exit points (asterisks). (B) A *turnout* CaP motor neuron projecting rostrally within the spinal cord, and exiting from an adjacent hemisegments midsegmental exit point. (D) A MiP motor neuron failing to migrate upon exit from the spinal cord. (F) A RoP motor axon projecting rostrally and exiting from an adjacent hemisegment exit. (G, H) *turnout* CaP and RoP motor neurons with correct axonal trajectories and ectopic branches. Dashed line indicates somite boundary. Scale bar, 10 µm.

To further define the role of the *turnout* gene in motor axon development, we examined the trajectories of individually labeled motor neurons. In wild-type embryos, primary motor axon outgrowth begins around 16 hpf by the sequential outgrowth of three main subtypes of primary motor neurons: Caudal Primary (CaP), Middle Primary, (MiP) and Rostral Primary (RoP) [3]. The axonal trajectories of each motor neuron subtype can be visualized by stochastic labeling using the *mnx1:GFP* construct, and each motor neuron subtype can be uniquely identified based on soma position within the spinal cord [20]. Importantly, analysis of markers specific for each motor neuronal subtype [21], [22] confirmed that the cell body positions of *turnout* mutant CaP, MiP and RoP motor neurons are unaffected (*islet1*, *islet2*; data not shown). In wild-type embryos CaP, MiP and RoP motor axons first navigate to the spinal cord exit point at the center of each hemisegment, and then pioneer into the ventral, dorsal and lateral myotome, respectively, initially without forming permanent peripheral branches (Fig. 2A, C, E). Overall, 24.6% (17/69) of individually labeled CaP, MiP and RoP *turnout* motor neurons displayed one of three intraspinal pathfinding defects. First, instead of projecting caudally towards the segmental exit point, *turnout* motor axons projected rostrally, and exited into the myotome through the segmental exit point in an adjacent hemisegment (Fig. 2B, F; n = 9/17). Second, mutant motor axons navigated properly to the endogenous exit point but then failed to exit from the spinal cord or stall shortly after exiting (Fig. 2D; n = 6/17). Third, mutant motor axons exited the spinal cord at ectopic, non-exit point location (Fig. 1B, open arrowhead; n = 2/17).

In the periphery, primary motor axons initially share part of their trajectory, and thus the axonal phenotype observed in *turnout* might reflect either a defasiculation or a branching defect. Analysis of individually labeled *turnout* motor neurons revealed that the abnormal peripheral axon morphology we had observed in the anti-synaptotagmin staining (Fig. 1B), is due to branching rather than reduced axonal fasciculation (Fig. 2G, H). Thus, in addition to controlling intraspinal motor axon guidance, the *turnout* gene is critical for peripheral branch morphogenesis.

### The *turnout* Gene acts Cell Non-autonomously

**Figure 3:**
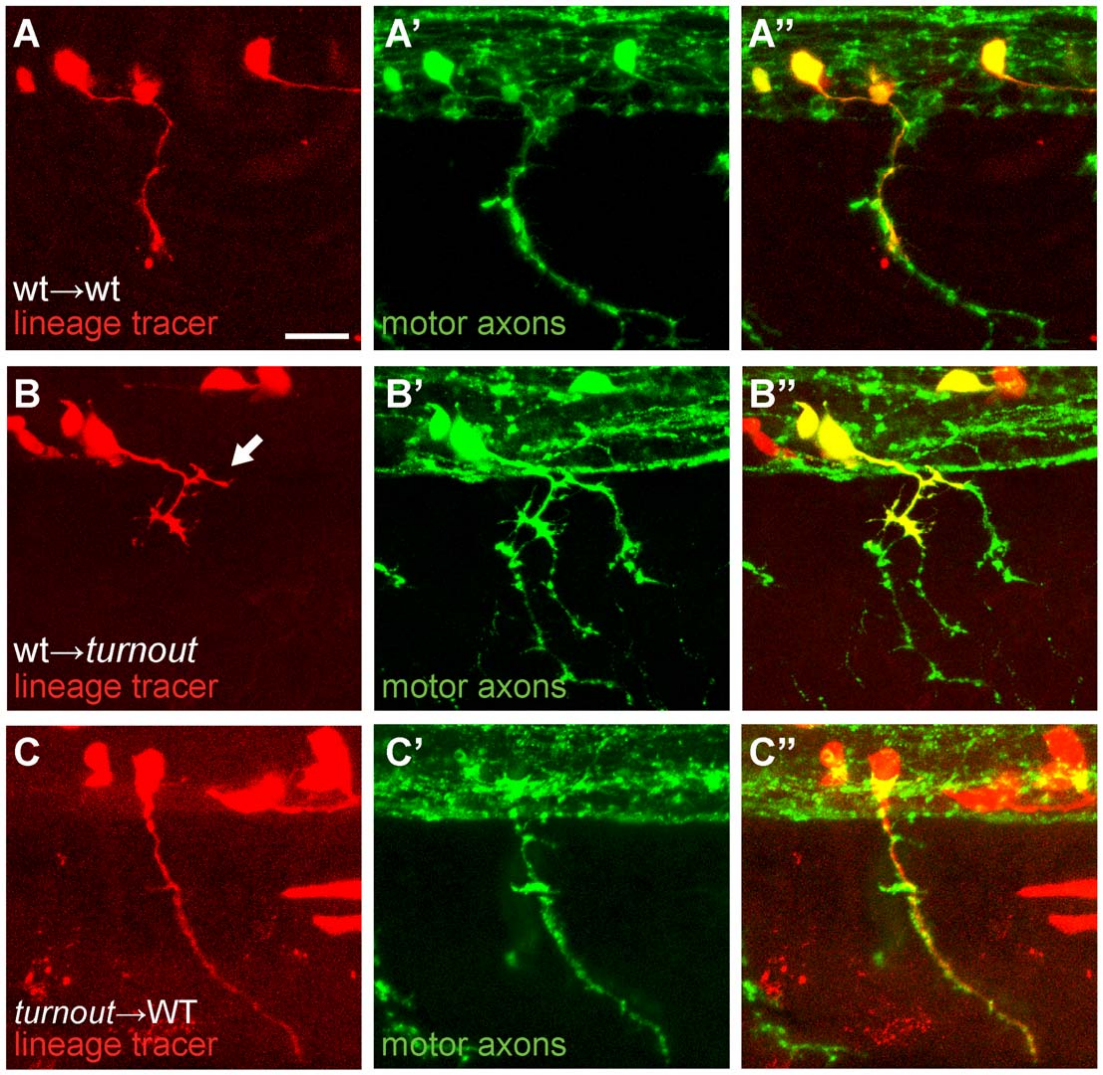
The *turnout* gene acts cell non-autonomously. (A, B, C) Donor cells are labeled with rhodamine dextran (red), and host embryos were stained with SV2 antibody (green) to reveal all motor axonal trajectories. (A) Wild-type donor-derived neurons (red) in a wild-type host display no guidance or branching defects (0/20). (B) Wild-type donor-derived neurons in a mutant host display precocious branching upon exiting the spinal cord (arrow, 9/18). (C) Mutant donor-derived motor axons display no defects in a wild-type host environment (0/6). Scale bar, 15 µm.

To determine the biological mechanism through which the *turnout* gene controls motor axon guidance and branching we investigated in which cell type(s) the *turnout* gene functions. For this, we generated chimeric embryos by transplanting wild-type blastula cells, uncommitted regarding their cell fate and labeled with a lineage tracer, into age-matched mutant hosts, and vice versa. Analysis of the chimeras at 24 hpf revealed that wild-type derived motor neurons displayed no guidance or branching errors when transplanted into wild-type hosts (Fig. 3A, n = 20/20). In contrast, analysis of *turnout* mutant host embryos revealed that 50% of wild-type derived motor neurons (Fig. 3B, n = 9/18) displayed characteristic *turnout-*like axon branching and guidance defects, consistent with the frequency of affected hemisegments in *turnout* mutant embryos (56%, Fig 1). Conversely, *turnout* mutant motor neurons when navigating through a wild-type host environment always displayed wild-type axonal trajectories (Fig. 3C, n = 6/6). Together, these results demonstrate that *turnout* functions cell non-autonomously for intraspinal motor axon guidance and for peripheral branch morphogenesis. Given the robustness of the peripheral branching phenotype, present in 42% of all somitic hemisegments (Fig. 1B), we next decided to examine in more detail the spatiotemporal process of motor axon branch morphogenesis.

### CaP Axons Form Branches First through Bifurcation and then through Collateral Branch Formation

**Figure 4:**
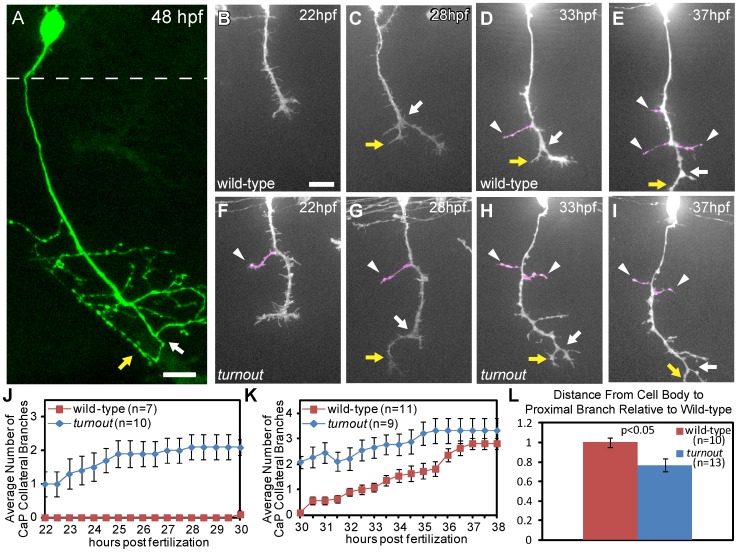
The *turnout* gene controls where and when collateral CaP axon branches form. (A) 48 hpf wild-type embryo with a single *mnx1:GFP* positive CaP motor axon. Note that the axon is devoid of branches along the proximal axon shaft, and that collateral branches only form along the distal axon segment, adjacent to the bifurcation branch (white arrow) with one branch turning rostrally (yellow arrow) and one caudally. Dashed line indicates spinal cord boundary. (B, C) Time-lapse of wild-type CaP axons traversing the ventral myotome between 22 hpf and 30 hpf. (D, E) In wild-type embryos, branching begins at ∼30 hpf, typically by axon bifurcation (arrow), followed by the formation of axon collateral branches (arrowhead). (F, G) *turnout* CaP axons form precocious collateral branches (arrowhead). (H, I) *turnout* axon collateral branching plateaus between 30 hpf and 38 hpf. (J) Quantification of CaP axon branches between 22–30 hpf. (K) Quantification of CaP axon branches between 30–38 hpf. (L) Quantification of the location of the first proximal collateral relative to wild-type at 48 hpf. Branches are defined as protrusions longer than 5 µm that persist for longer than 2 hours. Error bars indicate SEM. Scale bar, 15 µm.

During development, motor axons form specific peripheral branching patterns to innervate defined muscle fibers. Individual neurons can form multiple types of axonal branches, which is particularly evident in the CaP subtype of zebrafish motor neurons. CaP motor neurons invariantly innervate a specific subset of muscle fibers in the ventral myotome [3], [23]. By 48 hpf individual CaP axons have formed a single bifurcation branch at the ventral myotome, and approximately four primary collateral branches along the distal axon shaft, leaving the initial segment of the axon devoid of branches (Fig. 4A) [23]. CaP branching morphology has been shown to be conserved between body segments, between individual animals, and to persist into adulthood [3],[24], yet the dynamic process by which the bifurcational and collateral branches form has not been examined in live, intact animals.

To examine the dynamics of branch formation in live, intact zebrafish, we performed live cell imaging of individual CaP axons, stochastically labeled with a combination of cytoplasmic (*mnx1:GFP)* and membrane-tagged GFP (*mnx1:mCD8-GFP*) [20], [25]. Based on previous studies [3], [23], [24], we decided to image the emergence and location of axonal protrusions between 22 and 38 hpf. Between 22 and 30 hpf axonal protrusions up to 15 µm in length emerged along the axon shaft (Fig. 4B, C). These protrusions were transient and persisted for less than 90 minutes, consistent with previous reports that CaP axons do not form stable branches before reaching the ventral extent of the myotome, around 28–30 hpf (Fig. 4B, C) [23]. Once CaP growth cones reached the ventral extent of the myotome, they formed a characteristic bifurcation 72.9+/−5.2 µm from the cell body, with one branch turning caudally (Fig 4A, D, E), and a second branch turning rostrally towards the segmental border, eventually projecting dorsally to innervate the myoseptal boundary [26]–[28].

Beginning at 30 hpf, we detected the formation of the first stable CaP axons collateral branches, defined as protrusions along the axon shaft greater than 5 µm, that persist for longer than two hours (Fig. 4D). Over the course of our analyses, up to 5 days post fertilization, these protrusions were never observed to retract, and formed permanent stable branches. Thus, we used these criteria to predict when a protrusion will develop into a stable permanent branch. By 38 hpf, individual CaP axons had developed approximately three collateral branches, and by 48 hpf they formed 4.9+/−0.4 branches (n = 11; Fig. 4D, E, J, K). Importantly, collateral branches were only added to the distal portion of the axon shaft, resulting in a ‘branch free’ zone between the cell body and the first branch (Fig. 4A, D, E). Thus, CaP axons develop stereotypic and stable peripheral branches through a series of precisely timed and spatially restricted branching events.

### *turnout* Regulates CaP Axon Collateral Branch Morphogenesis

To determine if bifurcation and/or collateral branching is affected in *turnout* mutants, we examined the onset and locations of CaP axonal protrusion using live cell imaging, as outlined above. Both the location and the timing of growth cone bifurcation, which gives rise to the first axonal branch, was indistinguishable between wild-type siblings and *turnout* mutant CaP axons (arrows in Fig. 4C, D, E, G, H, I; quantified in Table 1), demonstrating that *turnout* is dispensable for CaP bifurcational branching. In contrast, collateral branch formation was severely affected in *turnout* mutants. Unlike wild-type sibling CaP axons, which are devoid of collateral branches prior to 30 hpf, *turnout* mutant CaP axons developed 2.1+/−0.2 stable axonal branches between 22 and 30 hpf (n = 10; Fig. 4F, G, J). Over the next 8 hours, the formation of new collateral branches plateaued (Fig. 4H, I, K), and by 48 hpf the total number of *turnout* mutant collateral branches was the same as in wild-type siblings (wild-type = 3.6+/−0.3 branches, n = 12; *turnout = *3.8+/−0.4 branches, n = 12).

**Table 1:** Quantification of axon branching defects in *turnout* and *plexin A3* mutants.

	*turnout*siblings	*turnout*mutants	*plexin A3* siblings	*plexin A3* mutants
Time of ventral bifurcation	29.8+/−0.5 hpf (n = 12)	28.9+/−0.3 hpf (n = 15)	29.4+/−0.3 hpf (n = 12)	29.2+/−0.4 hpf (n = 14)
Position of ventral bifurcation*	89.8+/−3.1 µm (n = 12)	93+/−3 µm (n = 15)	79.4+/−3.7 µm (n = 11)	84.0+/−2.4 µm (n = 13)
**Collateral branches at 30 hpf**	**0.08+/−0.08 (n = 7)**	**2.1+/−0.2 (n = 10)**	**0.12+/−0.08 (n = 17)**	**2.7+/−0.4 (n = 14)**
Collateral branches at 48 hpf	3.6+/−0.3 (n = 12)	3.8+/−0.4 (n = 12)	3.9+/−0.4 (n = 11)	3.4+/−0.3 (n = 12)
**Proximal branch position** *	**1.0+/−0.05 µm (n = 10)**	**0.77+/−0.07 µm (n = 13)**	**1.0+/−0.06 µm (n = 17)**	**0.53+/−0.04 µm (n = 12)**
**Total protrusions extended 22–30** **hpf**	**53.5+/−4.5 (n = 12)**	**22.4+/−3.1 (n = 10)**	**60.9+/−8.3 (n = 12)**	**34.1+/−3.0 (n = 12)**
**Rate of forming protrusion 22–30hpf** #	**7.4+/−0.7 (n = 12)**	**4.2+/−0.5 (n = 10)**	**8.6+/−1.2 (n = 12)**	**3.8+/−0.16 (n = 12)**
Ratio of proximal to distal protrusions§	0.39+/−0.2 (n = 12)	0.35+/−0.03 (n = 9)	0.41+/−0.03 (n = 12)	0.41+/−0.02 (n = 12)
**% protrusions that develop into** **branches 22–38 hpf**	**5.0+/−0.5% (n = 12)**	**9.7+/−1.4% (n = 9)**	**4.6+/−0.8% (n = 12)**	**12.9+/−2.1% (n = 12)**

* Relative Position to wild-type; measured as distance from the cell body to the branch point.

§ Proximal protrusions were scored if they were located between the cell body and the horizontal myoseptum; distal protrusions were scored if they were located between the horizontal myoseptum and the growth cone.

# Number of protrusions/hour.

To further explore how *turnout* controls peripheral branch formation, we asked whether *turnout* influences branch location. In *turnout* wild-type siblings the most proximal branch forms indistinguishable from those in wild type embryos, along the distal portion of their axons (1.0+/−0.05 relative to the average in wild-type axons, Fig. 4A, L; n = 10). In contrast, *turnout* mutant CaP axons formed collateral branches at significantly more proximal locations, the most proximal branch was 0.77+/−0.07 of the distance relative to average (Fig. 4L, n = 13). Thus, *turnout* is dispensable for controlling CaP bifurcation and regulating the total number of axon branches, but plays a specific role in controlling when and where collateral branches form.

### The Plexin A3 Guidance Receptor Regulates CaP Collateral Branch Formation

**Figure 5:**
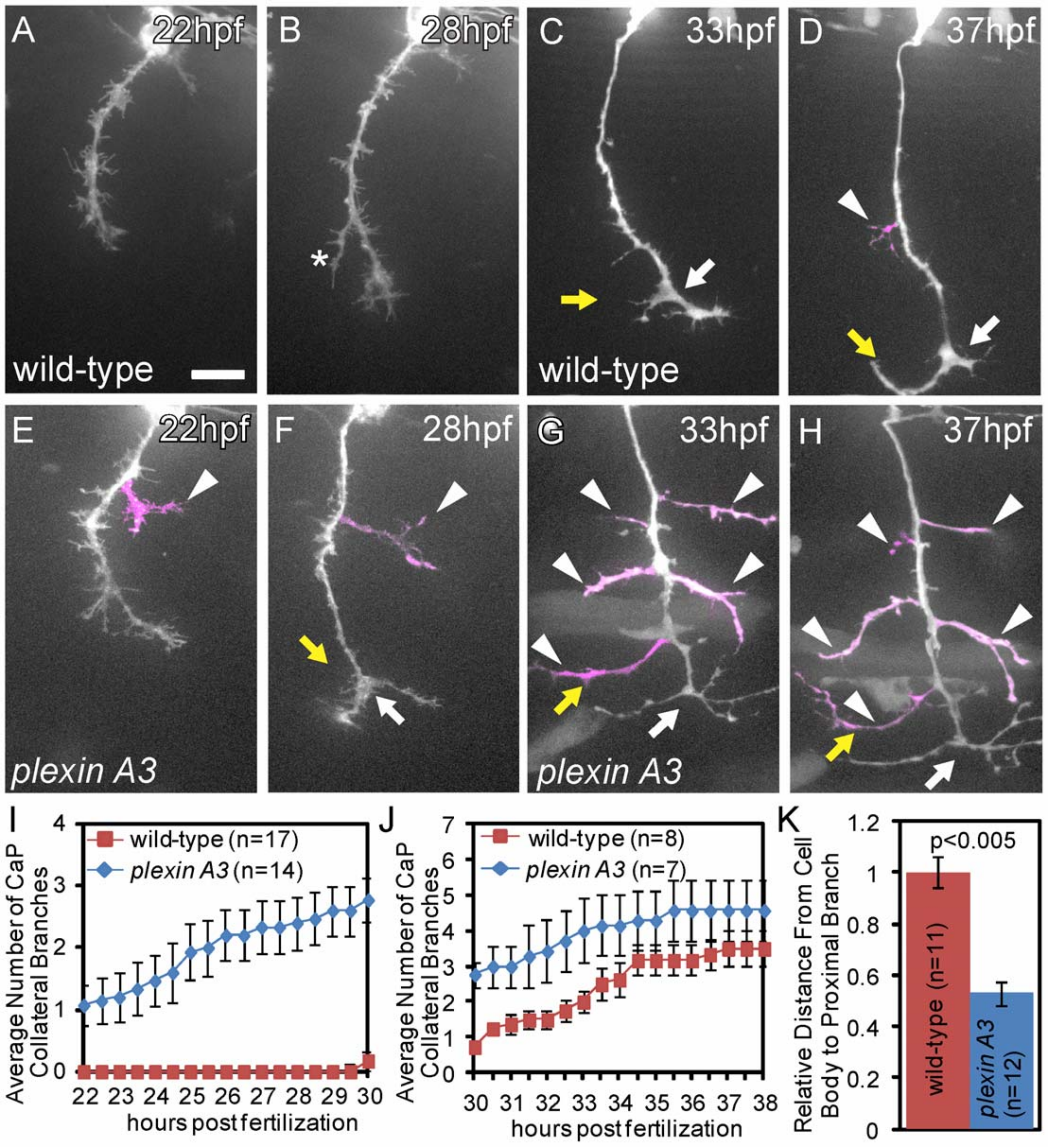
Plexin A3 controls where and when collateral CaP axon branches form. (A, B) Time-lapse of a wild-type CaP axon traversing the ventral myotome between 22 hpf and 30 hpf. (C, D) Time-lapse of a wild-type sibling embryo, demonstrating that branching begins at ∼30 hpf, typically with axonal bifurcation (arrow) followed by axon collateral formation (arrowhead). (E, F) In *plexin A3* mutants, collateral branches (arrowhead) form precociously between 22 and 30 hpf. (G, H) In *plexin A3* mutants collateral formation plateaus between 30 hpf and 38 hpf. (I) Quantification of CaP primary axon branches between 22–30 hpf. (J) Quantification of CaP primary axon branches between 30–38 hpf. (H) Quantification of the spatial location of the first proximal collateral relative to wild-type at 48 hpf. Error bars indicate SEM. Scale bar, 15 µm.

We had previously reported on the isolation of presumptive null mutations in the *plexin A3* guidance receptor [18]. At 24 hpf, *plexin A3* mutants display intraspinal motor axon guidance defects, as well as excessive peripheral motor axon branching, however its specific role in controlling branching had not been examined [20]. The similarity of the overall motor axonal defects between *turnout* and *plexin A3* mutants prompted us to examine in more detail the role of *plexin A3* during collateral branch morphogenesis. Live cell analysis to determine the onset and locations of CaP axonal protrusion in *plexin A3* mutants revealed dramatic defects in collateral branch formation. Similar to *turnout* mutants, CaP axons formed ectopic and precocious, permanent branches, almost immediately after exiting from the spinal cord (Fig. 5A–D, E–H). By 30 hours of development *plexin A3* CaP axons had formed on average 2.7+/−0.4 branches (n = 14; Fig. 5I), in the proximal portion of the axon (Fig. 5E, F). Between 30 and 38 hpf, when collateral branch formation occurs in wild-type CaP axons, branching declined in *plexin A3* mutants resulting in equal numbers of permanent collaterals formed by 48 hpf (Fig. 5G, H, J; quantified in Table 1). Additionally, collateral branches formed significantly more proximal to the cell body relative to wild-type (Fig. 5K; quantified in Table 1). In contrast, the timing and position of bifurcational branching was unaffected in *plexin A3* mutants (Table 1). Thus, similar to *turnout*, *plexin A3* is dispensable for CaP bifurcation and for regulating the total number of axonal branches, but plays a critical role in controlling when and where collateral branches form. Given the very similar defects in CaP collateral branch morphogenesis, we asked whether *plexin A3* and *turnout* function in a common genetic pathway. However, embryos heterozygous for both *plexin A3* and *turnout* did not display branching defects (data not shown), providing no conclusive result as to whether both genes act in a common or in parallel pathways.

### *turnout* and *plexin A3* Control Branching by Stabilizing Short-lived Axonal Protrusions

Axonal branching is a highly regulated cellular process that is the result of the frequency by which protrusions are generated, their growth rate, and the rate by which they become stable branches. Given the branching phenotype observed in *turnout* and *plexin A3* mutants, one possible mechanism by which these genes might regulate collateral branch morphogenesis is by suppressing the initiation of axonal protrusions during the time period when the growth cones still traverses the ventral myotome. Alternatively, *turnout* and *plexin* A3 might function to suppress the transition of transient protrusions into stable branches during this time period. To determine which aspects of branch formation *plexin A3* and *turnout* regulates, we used live cell imaging and measured the initiation frequency of transient protrusions (>5 µm in length), their locations, and the transition rate of protrusions into stable branches. As outlined below, both *plexin A3* and *turnout* promote the formation of transient axonal protrusions, while they also suppress the transition from early protrusions to stable branches.

**Figure 6:**
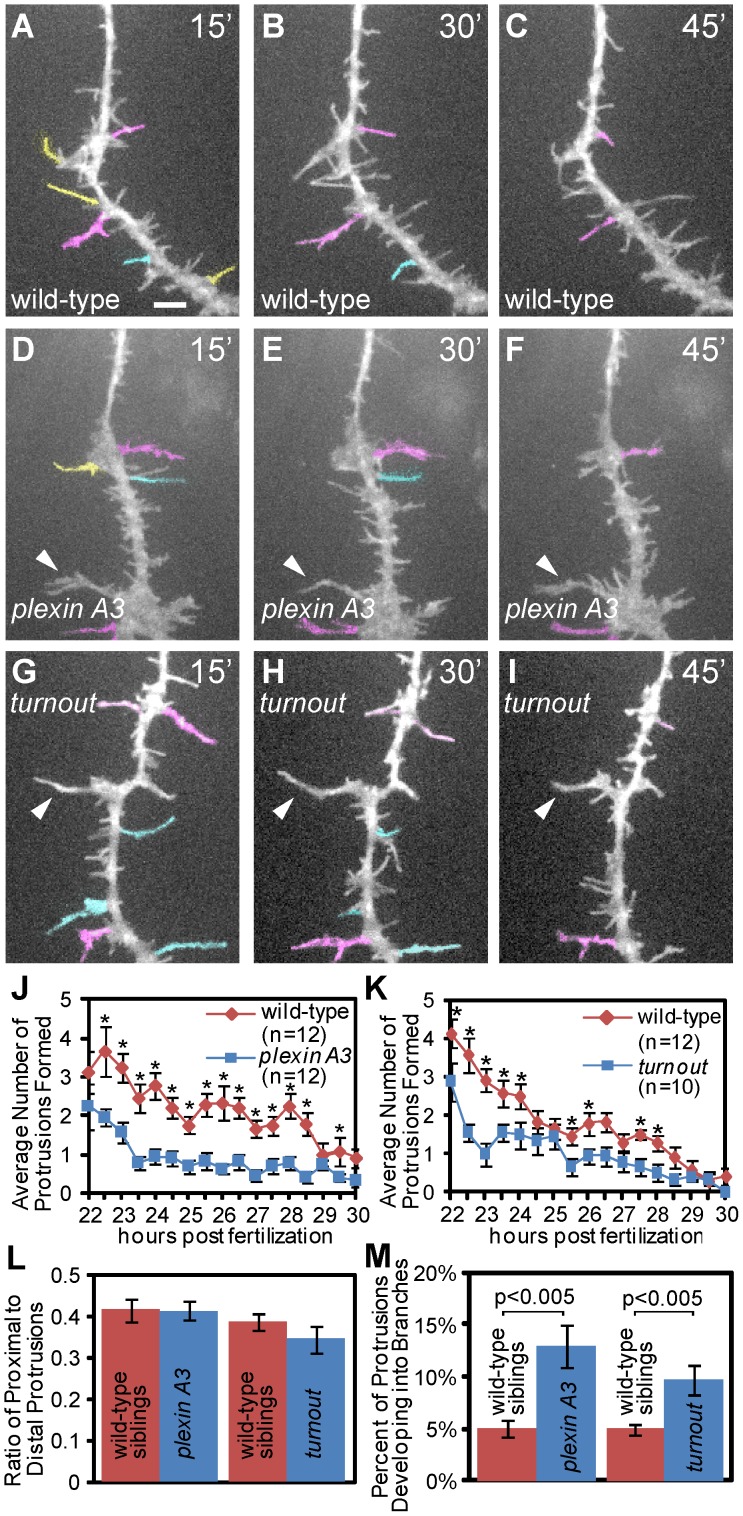
Between 22 and 30 hpf *turnout* and *plexin A3* mutant axons initiate fewer short term protrusions but stabilize more into branches. (A, B, C) Representative still images from a time lapse movie of a wild-type CaP axon (D, E, F) Representative still images from a time lapse movie of a *plexinA3* mutant CaP axon. (G, H, I) Representative still images from a time lapse movie of a *turnout* mutant CaP axon. Yellow: protrusions greater than 5 µm with a lifetime of less than 30 minutes. Cyan: protrusions with a lifetime of 30–45 minutes. Magenta: protrusions with a lifetime of 45–60 minutes. Arrowheads indicate stable, permanent branches. (J) Quantification of the average number of new *plexin A3* mutant protrusions formed over time. (K) Quantification of the average number of new *turnout* mutant protrusions formed over time. (L) Quantification of the ratio of proximal to distal protrusions formed indicates the location of protrusions extended. (M) Percentage of protrusions that develop into branches. Error bars indicate SEM. Scale bar, 5 µm.

Specifically, between 22 and 30 hpf, individual CaP axons in *plexin A3* wild-type siblings (n = 12) or *turnout* wild-type siblings (n = 12) form a total of 60.9+/−8.3 and 53.5+/−4.5 protrusions, respectively, at a rate of 8.6+/−1.2 and 7.4+/−0.7 protrusions per hour, respectively (Fig. 6 A-K). In wild-type, these protrusions are transient, and the majority of these protrusions, 91.4+/−1.2% for *plexin A3* wild-type siblings and 91.0+/−1.5% for *turnout* siblings, have a lifespan of less than 30 minutes (Fig. 6A, yellow protrusions). In contrast, both *plexin A3* and *turnout* CaP axons form significant fewer protrusions (*plexin A3∶*34.1+/−3.0 p<0.005; *turnout*: 22.4+/−3.1, p<0.005), due to significantly reduced rates of 3.8+/−0.16 (n = 12) and 4.2+/−0.5 (n = 9) of protrusions per hour, respectively (Fig. 6D–K). Thus, despite the formation of stable, ectopic collateral branches in *plexin A3* and *turnout* mutants, the rate with which transient axonal protrusions are initiated is reduced, suggesting that both genes function to promote the initiation of transient axonal protrusions while suppressing stable branch formation. Importantly, neither *plexin A3* nor *turnout* mutants displayed defects in the location of these transient protrusions (Fig. 6L).

Finally, we examined the fraction of early protrusions that eventually developed into permanent branches. In CaP axons of *plexin* A3 and *turnout* wild-type siblings, an average of 4.6+/−0.8% (n = 12) and 5.0+/−0.5% (n = 12) of all protrusions (>5 µm in length) developed into permanent branches, respectively. In contrast, *plexin A3* CaP axons displayed a 2.8 fold increase in the percentage of protrusion that transitioned into permanent branches (12.9+/−2.1%, n = 12; p<0.005, Fig. 6M). Similarly, *turnout* mutant CaP axons exhibited an almost 2 fold increase of protrusion that transitioned into permanent branches (9.7+/−1.4%, n = 9; p<0.005, Fig. 6M). Thus, both *plexin A3* and *turnout* also function to suppress the transition from early protrusions to stable branches. Combined, our analyses show that *plexin A3* and *turnout* modulate two aspects of collateral branch formation between 22 and 30 hpf, when CaP growth cones pioneer into and through the ventral myotome. First, both genes promote transient axonal protrusions, which might aid in growth cone guidance. Consistent with this, *plexin A3* and *turnout* mutant CaP axons display guidance defects (Fig. 2) [20]. Second, *plexin A3* and *turnout* suppress precocious and ectopic branch formation by destabilizing transient protrusions, thereby ensuring that collateral branches form only after the growth cone has traversed the myotome, and only in the distal portion of the axon shaft. Thus, live cell imaging reveals a specific role for *plexin A3* and *turnout* regulating axonal protrusions.

## Discussion

The *in vivo* mechanisms that regulate when and where axons generate protrusions, extend them and then convert them into stable branches is not well understood. Taking advantage of its amenability for genetic approaches and live cell imaging of individual neurons, we focus here on an identified motor neuron, CaP, to identify genes and mechanism that regulate its intricate axonal branching pattern. Our results reveal that CaP axons develop stereotypic and stable peripheral branches through a series of precisely timed and spatially restricted branching events. Moreover, we identify two genes, *turnout* and *plexin A3* that are dispensable for controlling CaP bifurcation and regulating the total number of axon branches, and instead play specific roles in controlling *when* and *where* collateral branches form. Finally, we find that both genes have two apparently opposing effects during collateral branch formation- promoting transient axonal protrusions while destabilizing transient protrusions. We propose that both genes contribute to a mechanism that balances a migrating axon’s requirements to promote transient axonal protrusions critical for growth cone motility while suppressing precocious and ectopic branch formation to ensure that collateral branches only form *when* and *where* they are appropriate.

### The *turnout* Mutation Affects Motor Axon Guidance and Collateral Branching

We mapped the *turnout* locus to a ∼300 Kb region containing eight annotated open reading frames, however, none of these genes harbored nonsense mutations in the DNA and mRNA isolated from *turnout* mutants (Fig. 1). Based on chimera analyses (Fig. 3), we predict the *turnout* gene to be dispensable in motor neurons and instead function in the embryonic environment. This would eliminate most of the eight genes and might favor *integrin beta 1a* as the candidate gene for *turnout*. Traditionally, beta integrins are thought to function cell autonomously within neurons and to bind to ECM components, such as laminin or tenacin-C, thereby mediating axon outgrowth [29]–[31]. However, integrins have recently also been shown to function non cell-autonomously for motor axon guidance. For example, the muscle specific, but not the motor neuron specific, knockout of *integrin beta 1* causes spinal motor axons overshoot their target muscle and to branch excessively [32], somewhat reminiscent of the *turnout* phenotype. In zebrafish, *integrin beta 1a* mRNA is expressed between 2 and 96 hpf, and thus during the time period of motor axon guidance and branching [33], however, its spatial expression pattern has not been examined. Given that missense and nonsense mutations are absent in *turnout integrin beta 1a* mRNA, it is possible that the *turnout* phenotype is due to a mutation in non-coding, regulatory regions of the gene, causing altered gene expression. Future studies, starting with complete sequencing of the 300 kb interval will be required to definitively identify the molecular nature of the *turnout* gene.

### *plexin A3* Regulates the Timing and Location of Motor Axon Collateral Branches

*In vivo* time-lapse microscopy has proven invaluable in revealing critical steps during axon and dendritic branch morphogenesis, revealing for example how contact mediated repulsion is predominantly responsible for the spatially self organizing branching of trigeminal axon arbors [34]. Using time-lapse microscopy of individual CaP motor neurons *in vivo*, we find that the guidance receptor *plexinA3* regulates the timing and location of collateral axon formation, without affecting total branch number (Fig. 5). *plexin A3* belongs to a family of neuronal receptors that mediate semaphorin signaling to regulate axonal guidance but also synapse formation and axonal pruning [35]. For example, mouse embryos lacking *plexin A4 or plexin A3 and plexin A4* exhibit extensive defasciculation and sprouting of cranial and spinal nerves, and overshooting of ophthalmic nerve fibers [36], [37]. At post-natal ages, Plexin A3 and Plexin A4 receptors mediate elimination of synaptic complexes and axonal pruning [38], [39].

Recently, Plexin A receptor function has also been shown to act specifically in shaping axonal branching morphology of *Drosophila* mechanosensory neurons [40]. There, RNAi mediated reduction of *plexinA* increased the axonal arbor complexity by increasing branch length and the total number of branches, without an apparent change in branch location. In contrast, we find that zebrafish CaP motor axons lacking *plexin A3* exhibit a change in branch location without alterations in the total number of branches (Fig. 5). Interestingly, such change in branch morphology, independent of changes in total branch numbers, is observed in cultured hippocampal neurons lacking *kinesin 2A* [41], [42]. There, cultures neurons exhibit increased collateral length without altering the total number of branches, and *in vivo* single cell labeling of the *kinesin 2A* hippocampal neurons revealed branches along ectopic axonal locations [41], [42]. Independent of the precise mechanisms by which *Plexin A3* regulates branch morphogenesis, our studies provide compelling evidence that, at least in zebrafish motor neurons, mechanisms that regulate branch location can act independently of those controlling total branch number.

Analysis of CaP branch morphogenesis in *plexin A3* mutants reveals that collateral branches form at ectopic positions as they begin to form immediate after CaP axons exit the spinal cord and traverses the ventral somite. Our data is consistent the idea that *where* collateral branches form is determined by the spatially restricted expression of semaphorin ligands along the path of the CaP axon towards the ventral somite. In fact, several *sema3* ligands, including *sema3aa* are expressed in the myotome [43]. In wild-type CaP axons this *plexin A3* dependent mechanism ensures that collateral branching only occurs once the growth cones reaches the ventral extent of the somite, leading to the formation of collateral branches restricted to the area adjacent to the growth cone. In contrast, loss of semaphorin sensitivity in *plexin A3* mutant CaP axons results in the production of collateral branches as soon as the growth cones begin to migrate though the somite (Fig. 5).

Finally, loss of semaphorin sensitivity in *plexin A3* motor axons might also account for axonal guidance defects. In fact, we had previously shown that during their intraspinal migration, *plexin A3* acts cell autonomously to direct RoP and MiP motor growth cones towards the segmental exit point, and proposed that this is due to repulsive *plexin A3* signaling, possibly triggered by semaphorins secreted from the posterior somite territory [20]. Although it is tempting to speculate that different semaphorin ligands might control growth cone guidance and branch morphology via a common *plexin A3* receptor, identification of the relevant ligands and the relevant downstream signaling pathways is required to determine if and to which extent the processes of *plexin A3* dependent guidance and branching overlap.

### *turnout* and *plexin A3* Induce Axonal Protrusions while Inhibiting Branch Formation

Use of time-lapse microscopy in cell culture has revealed a stepwise progression of protrusion formation, extension and stabilization leading to the development of a mature axon branch [44]. Our analyses demonstrate that while wild-type CaP motor axons traverse the ventral myotome they form many short-term protrusions, but no branches (Fig. 1). Once the first collateral branches begin to form, the number of these short-lived protrusions decreases (Fig. 6). In *turnout* and *plexin A3* mutants, protrusions form at a significantly lower rate, resulting in fewer short-term protrusions, while protrusions are more likely stabilize into branches (Fig. 6).

These data indicate a dual role for *turnout* and *plexin A3* to encourage the formation of short-term protrusions while suppressing stable branch formation. One possibility for this seemingly contradictory data is that during axon outgrowth, CaP motor axons are in an exploratory state, where it is beneficial to generate more *plexinA*3 and *turnout* dependent, short-term membrane protrusions, critical for proper growth cone navigation. Consequently, loss of *plexin A3* or *turnout* leads to significant motor axon guidance defects (Fig. 1) [20]. At the same time, more *plexin A*3 and *turnout* also inhibit the transition of membrane protrusions to stable branches, thereby ensuring that collateral branches only form once the growth cone has traversed the ventral myotome. Thus, our data reveals a CaP motor neuron intrinsic role for *plexin A3* and an extrinsic role for *turnout* in regulating the formation of membrane protrusions and in regulation their stability.

How does PlexinA3 signaling regulate the formation of membrane protrusions and their stability? Exposure of Semaphorin 3A to cultured hippocampal neurons inhibits the formation of new protrusions and destabilizes branches [14], only partially consistent with the defects we observe in *plexin A3* mutant CaP axons. While the relevant signaling events downstream of *plexin* signaling that mediate axonal branching are currently unknown, it is well documented that *plexin* signaling at the growth cone leads to actin depolymerization through several mechanisms, either directly through proteins such as MICAL or indirectly through activation of Rho family GTPases, key regulators of the actin cytoskeleton [45]–[47]. Whether similar downstream effectors regulate *plexin A3* dependent branch morphogenesis is an important question that will be the focus of future experiments.

## Materials and Methods

### Ethics Statement

All experiments were conducted according to an Animal Protocol fully approved by the University of Pennsylvania Institutional Animal Care and Use Committee (IACUC) on 4-06-2010, protocol number 801067. Veterinary care is under the supervision of the University Laboratory Animal Resources (ULAR) of the University of Pennsylvania.

### Fish Maintenance and Breeding

All experiments were performed with the *turnout^p5THBD^* and *plexin* ^p55emcf^ alleles. All wild-type fish were from either the Tübingen (Tü),Tupfel Long-Fin (TLF) or AB background. The *turnout* mutation was generated in an ENU mutagenesis screen in a mixed AB/Tü background as reported by [18].

### Immunohistochemistry

Embryos were fixed and stained as previously described in [48]. The primary antibodies and dilutions they were used at are as follows: znp-1 (1∶200, DSHB), SV2 (1∶50, DSHB), ZN12 (1∶50, DSHB), F59 (1∶20, DSHB), 4D9 (1∶2, DSHB), 3A10 (1∶50, DSHB), JL8 (1∶100,Clontech). Antibodies were visualized with corresponding Alexa-Fluor-488 or Alexa-Fluor-594 fluorescently conjugated secondary antibodies (1∶400; Molecular Probes, Eugene, OR). Embryos were mounted in Vectashield (Molecular Probes) and imaged with a Zeiss 510 confocal microscope.

### Single Cell Labeling

Embryos were injected with *mnx1:GFP* plasmid DNA (previously hb9:GFP) [49] alone, or in combination with *mnx1:mCD8-GFP* [25] at the single cell stage, and allowed to develop to 24 hpf. At this stage, they were fixed, and stained with the SV2 antibody as outlined above. To distinguish between the individual classes of motor neurons (CaP, MiP, RoP) we determined the ratio of the distance between exit points (a), and between the center of the soma and the endogenous exit point (b) as previously described in [20]. The ratios (a:b) for wild-type CaP/VaP motor neurons were always less than 0.1, for MiP (0.2–0.45) and RoP (0.3–0.62). Because of the slight variability in the positions of MiP and RoP motor neuron somas, only clearly categorized mutant motor neurons were scored. Motor neurons for with a ratio between 0.2–0.4 were scored as MiP, and ratios greater than 0.5 scored as RoP.

### Time Lapse Microscopy

Wild-type and mutant embryos transiently expressing the *mnx1*:GFP and *mnx1:mCD8-GFP* plasmids were anesthetized in 0.02% tricaine and embedded in 1.5% low-melting-point agarose. We only imaged motor axons projecting into somite 6–14 to minimize the developmental wave along the rostro-caudal axis. Time-lapse analysis was carried out on a spinning disk confocal microscope using a 63X water immersion lens equipped with a 28C environmental chamber. At 15-minute intervals, *z*-stacks of ∼30 µm were captured using Slidebook (3i) and then flattened by maximum projection. Images were further processed by adjusting the gamma levels using ImageJ and highlighting axon branches in Adobe Photoshop.

### Chimeric Analysis

Chimeric embryos were generated and analyzed as previously reported in [50]. Wild-type donor cells were from *Tg(mnx1:GFP)* transgenic embryos.

### Molecular Genetic Mapping

To map the *turnout^p5THBD^* mutation, phenotypically mutant embryos were first identified using SV2 antibody staining, and their genomic DNA isolated. The mapping procedure was performed as described in [51] using the following forward and reverse primers z5075∶5′-TGTGTTGTCTGATATGCCCACA-3′and 5′-TGCAAAAAGCCTCTAAGTGCCG-3′; CA447∶5′-TTCACACACAGATCCTGCTA-3′ and 5′-TTTTCAGTCCTTCAGAGAGC-3′; PIEZO2 intron 5′- CAGTACGCTGATATGGATGA-3′ and 5′-ACATGGACACTGACAATCCT-3′;nrp1a intron: 5′-TCTAACGAAGAGCTGTCCTC-3′ and GGCAGGATGACATGATAACT; z27195∶5′- GCAGATCCGGATGGAGTTTA-3′ and 5′- CACAGAATAGGCGTGGCTAA-3′. The amplification products were gel extracted, sequenced and then analyzed using Sequencher software.

## Supporting Information

Figure S1 **Muscle fiber development, muscle pioneer specification and position and extracellular matrix appear unaffected in** ***turnout*** **mutant embryos.** (A, B) Lateral view of 28 hpf wild-type. Scale bar, 25 µm. (A) and *turnout* (B) mutant embryos stained with the F59 antibody which recognizes myosin heavy chain in adaxial cell derived slow-twitch muscles cells indicates appropriate muscle fiber development and somite morphology. (C, D) Lateral view of 25 hpf wild-type (C) and *turnout* (D) embryos stained with the 4D9 antibody, which recognizes the nuclear Engrailed epitope in muscle pioneers indicates the correct number and position of pioneers cells present in each hemisegment located rostrally at the horizontal myoseptum. Scale bar, 20 µm. (E, F) Cross section of 24 hpf wild-type (E) and *turnout* (F) embryos stained with the chondroitin sulfate proteoglycan antibody indicates proper deposition of the extracellular matrix surrounding the spinal cord and the notochord. Scale bar, 25 µm.
(TIF)

## References

[1] Lichtman JW, Sanes JR (2003) Watching the neuromuscular junction. J Neurocytol 32: 767–775. 15034266 10.1023/B:NEUR.0000020622.58471.37

[2] Sanes JR, Lichtman JW (1999) Development of the vertebrate neuromuscular junction. Annu Rev Neurosci 22: 389–442. 10202544 10.1146/annurev.neuro.22.1.389

[3] Eisen JS, Myers PZ, Westerfield M (1986) Pathway selection by growth cones of identified motoneurones in live zebra fish embryos. Nature 320: 269–271. 3960108 10.1038/320269a0

[4] Bonanomi D, Pfaff SL (2010) Motor axon pathfinding. Cold Spring Harb Perspect Biol 2: a001735. 20300210 10.1101/cshperspect.a001735 PMC2829954

[5] Andlauer TF, Sigrist SJ (2012) In vivo imaging of the Drosophila larval neuromuscular junction. Cold Spring Harb Protoc 2012: 481–489. 22474664 10.1101/pdb.prot068593

[6] Wyatt RM, Balice-Gordon RJ (2003) Activity-dependent elimination of neuromuscular synapses. J Neurocytol 32: 777–794. 15034267 10.1023/B:NEUR.0000020623.62043.33

[7] Gibson DA, Ma L (2011) Developmental regulation of axon branching in the vertebrate nervous system. Development 138: 183–195. 21177340 10.1242/dev.046441 PMC3005597

[8] Dent EW, Kalil K (2001) Axon branching requires interactions between dynamic microtubules and actin filaments. J Neurosci 21: 9757–9769. 11739584 10.1523/JNEUROSCI.21-24-09757.2001 PMC6763027

[9] Yu W, Ahmad FJ, Baas PW (1994) Microtubule fragmentation and partitioning in the axon during collateral branch formation. J Neurosci 14: 5872–5884. 7931550 10.1523/JNEUROSCI.14-10-05872.1994 PMC6576981

[10] Grider MH, Park D, Spencer DM, Shine HD (2009) Lipid raft-targeted Akt promotes axonal branching and growth cone expansion via mTOR and Rac1, respectively. J Neurosci Res 87: 3033–3042. 19530170 10.1002/jnr.22140 PMC6561343

[11] Gallo G, Letourneau PC (1998) Localized sources of neurotrophins initiate axon collateral sprouting. J Neurosci 18: 5403–5414. 9651222 10.1523/JNEUROSCI.18-14-05403.1998 PMC6793492

[12] Schaefer AM, Hadwiger GD, Nonet ML (2000) rpm-1, a conserved neuronal gene that regulates targeting and synaptogenesis in C. elegans. Neuron 26: 345–356. 10839354 10.1016/s0896-6273(00)81168-x

[13] Wang KH, Brose K, Arnott D, Kidd T, Goodman CS, et al (1999) Biochemical purification of a mammalian slit protein as a positive regulator of sensory axon elongation and branching. Cell 96: 771–784. 10102266 10.1016/s0092-8674(00)80588-7

[14] Dent EW, Barnes AM, Tang F, Kalil K (2004) Netrin-1 and semaphorin 3A promote or inhibit cortical axon branching, respectively, by reorganization of the cytoskeleton. J Neurosci 24: 3002–3012. 15044539 10.1523/JNEUROSCI.4963-03.2004 PMC6729836

[15] Panagiotaki N, Dajas-Bailador F, Amaya E, Papalopulu N, Dorey K (2010) Characterisation of a new regulator of BDNF signalling, Sprouty3, involved in axonal morphogenesis in vivo. Development 137: 4005–4015. 21062861 10.1242/dev.053173 PMC2976284

[16] Barnes SH, Price SR, Wentzel C, Guthrie SC (2010) Cadherin-7 and cadherin-6B differentially regulate the growth, branching and guidance of cranial motor axons. Development 137: 805–814. 20147381 10.1242/dev.042457 PMC2827690

[17] Taniguchi M, Yuasa S, Fujisawa H, Naruse I, Saga S, et al (1997) Disruption of semaphorin III/D gene causes severe abnormality in peripheral nerve projection. Neuron 19: 519–530. 9331345 10.1016/s0896-6273(00)80368-2

[18] Birely J, Schneider VA, Santana E, Dosch R, Wagner DS, et al (2005) Genetic screens for genes controlling motor nerve-muscle development and interactions. Dev Biol 280: 162–176. 15766756 10.1016/j.ydbio.2005.01.012

[19] Melancon E, Liu DW, Westerfield M, Eisen JS (1997) Pathfinding by identified zebrafish motoneurons in the absence of muscle pioneers. J Neurosci 17: 7796–7804. 9315900 10.1523/JNEUROSCI.17-20-07796.1997 PMC6793908

[20] Palaisa KA, Granato M (2007) Analysis of zebrafish sidetracked mutants reveals a novel role for Plexin A3 in intraspinal motor axon guidance. Development 134: 3251–3257. 17699603 10.1242/dev.007112

[21] Appel B, Korzh V, Glasgow E, Thor S, Edlund T, et al (1995) Motoneuron fate specification revealed by patterned LIM homeobox gene expression in embryonic zebrafish. Development 121: 4117–4125. 8575312 10.1242/dev.121.12.4117

[22] Inoue A, Takahashi M, Hatta K, Hotta Y, Okamoto H (1994) Developmental regulation of islet-1 mRNA expression during neuronal differentiation in embryonic zebrafish. Dev Dyn 199: 1–11. 8167375 10.1002/aja.1001990102

[23] Myers PZ, Eisen JS, Westerfield M (1986) Development and axonal outgrowth of identified motoneurons in the zebrafish. J Neurosci 6: 2278–2289. 3746410 10.1523/JNEUROSCI.06-08-02278.1986 PMC6568750

[24] Liu DW, Westerfield M (1990) The formation of terminal fields in the absence of competitive interactions among primary motoneurons in the zebrafish. J Neurosci 10: 3947–3959. 2269893 10.1523/JNEUROSCI.10-12-03947.1990 PMC6570053

[25] Banerjee S, Gordon L, Donn TM, Berti C, Moens CB, et al (2011) A novel role for MuSK and non-canonical Wnt signaling during segmental neural crest cell migration. Development 138: 3287–3296. 21750038 10.1242/dev.067306 PMC3133918

[26] Downes GB, Granato M (2004) Acetylcholinesterase function is dispensable for sensory neurite growth but is critical for neuromuscular synapse stability. Dev Biol 270: 232–245. 15136152 10.1016/j.ydbio.2004.02.027

[27] Lefebvre JL, Jing L, Becaficco S, Franzini-Armstrong C, Granato M (2007) Differential requirement for MuSK and dystroglycan in generating patterns of neuromuscular innervation. Proc Natl Acad Sci U S A 104: 2483–2488. 17284594 10.1073/pnas.0610822104 PMC1892914

[28] Panzer JA, Gibbs SM, Dosch R, Wagner D, Mullins MC, et al (2005) Neuromuscular synaptogenesis in wild-type and mutant zebrafish. Dev Biol 285: 340–357. 16102744 10.1016/j.ydbio.2005.06.027

[29] Weaver CD, Yoshida CK, de Curtis I, Reichardt LF (1995) Expression and in vitro function of beta 1-integrin laminin receptors in the developing avian ciliary ganglion. J Neurosci 15: 5275–5285. 7542700 10.1523/JNEUROSCI.15-07-05275.1995 PMC2712128

[30] Varnum-Finney B, Venstrom K, Muller U, Kypta R, Backus C, et al (1995) The integrin receptor alpha 8 beta 1 mediates interactions of embryonic chick motor and sensory neurons with tenascin-C. Neuron 14: 1213–1222. 7541634 10.1016/0896-6273(95)90268-6 PMC2692383

[31] Tomaselli KJ, Reichardt LF (1988) Peripheral motoneuron interactions with laminin and Schwann cell-derived neurite-promoting molecules: developmental regulation of laminin receptor function. J Neurosci Res 21: 275–285. 2975342 10.1002/jnr.490210220

[32] Schwander M, Shirasaki R, Pfaff SL, Muller U (2004) Beta1 integrins in muscle, but not in motor neurons, are required for skeletal muscle innervation. J Neurosci 24: 8181–8191. 15371519 10.1523/JNEUROSCI.1345-04.2004 PMC6729792

[33] Mould AP, McLeish JA, Huxley-Jones J, Goonesinghe AC, Hurlstone AF, et al (2006) Identification of multiple integrin beta1 homologs in zebrafish (Danio rerio). BMC Cell Biol 7: 24. 16787535 10.1186/1471-2121-7-24 PMC1538996

[34] Sagasti A, Guido MR, Raible DW, Schier AF (2005) Repulsive interactions shape the morphologies and functional arrangement of zebrafish peripheral sensory arbors. Curr Biol 15: 804–814. 15886097 10.1016/j.cub.2005.03.048

[35] Waimey KE, Cheng HJ (2006) Axon pruning and synaptic development: how are they per-plexin? Neuroscientist 12: 398–409. 16957002 10.1177/1073858406292631

[36] Suto F, Ito K, Uemura M, Shimizu M, Shinkawa Y, et al (2005) Plexin-a4 mediates axon-repulsive activities of both secreted and transmembrane semaphorins and plays roles in nerve fiber guidance. J Neurosci 25: 3628–3637. 15814794 10.1523/JNEUROSCI.4480-04.2005 PMC6725384

[37] Yaron A, Huang PH, Cheng HJ, Tessier-Lavigne M (2005) Differential requirement for Plexin-A3 and -A4 in mediating responses of sensory and sympathetic neurons to distinct class 3 Semaphorins. Neuron 45: 513–523. 15721238 10.1016/j.neuron.2005.01.013

[38] Liu DY, Kuhlmey BT, Smith PM, Day DA, Faulkner CR, et al (2008) Reflection across plant cell boundaries in confocal laser scanning microscopy. J Microsc 231: 349–357. 18778432 10.1111/j.1365-2818.2008.02068.x

[39] Low LK, Liu XB, Faulkner RL, Coble J, Cheng HJ (2008) Plexin signaling selectively regulates the stereotyped pruning of corticospinal axons from visual cortex. Proc Natl Acad Sci U S A 105: 8136–8141. 18523013 10.1073/pnas.0803849105 PMC2430372

[40] Neufeld SQ, Hibbert AD, Chen BE Opposing roles of PlexinA and PlexinB in axonal branch and varicosity formation. Mol Brain 4: 15. 21489263 10.1186/1756-6606-4-15 PMC3094289

[41] Homma N, Takei Y, Tanaka Y, Nakata T, Terada S, et al (2003) Kinesin superfamily protein 2A (KIF2A) functions in suppression of collateral branch extension. Cell 114: 229–239. 12887924 10.1016/s0092-8674(03)00522-1

[42] Noda Y, Niwa S, Homma N, Fukuda H, Imajo-Ohmi S, et al (2012) Phosphatidylinositol 4-phosphate 5-kinase alpha (PIPKalpha) regulates neuronal microtubule depolymerase kinesin, KIF2A and suppresses elongation of axon branches. Proc Natl Acad Sci U S A 109: 1725–1730. 22307638 10.1073/pnas.1107808109 PMC3277188

[43] Sato-Maeda M, Tawarayama H, Obinata M, Kuwada JY, Shoji W (2006) Sema3a1 guides spinal motor axons in a cell- and stage-specific manner in zebrafish. Development 133: 937–947. 16452100 10.1242/dev.02268

[44] Gallo G (2010) The cytoskeletal and signaling mechanisms of axon collateral branching. Dev Neurobiol 71: 201–220. 10.1002/dneu.20852 21308993

[45] Hung RJ, Yazdani U, Yoon J, Wu H, Yang T, et al (2010) Mical links semaphorins to F-actin disassembly. Nature 463: 823–827. 20148037 10.1038/nature08724 PMC3215588

[46] Hung RJ, Pak CW, Terman JR (2011) Direct redox regulation of F-actin assembly and disassembly by Mical. Science 334: 1710–1713. 22116028 10.1126/science.1211956 PMC3612955

[47] Puschel AW (2007) GTPases in semaphorin signaling. Adv Exp Med Biol 600: 12–23. 17607943 10.1007/978-0-387-70956-7_2

[48] Zeller J, Schneider V, Malayaman S, Higashijima S, Okamoto H, et al (2002) Migration of zebrafish spinal motor nerves into the periphery requires multiple myotome-derived cues. Dev Biol 252: 241–256. 12482713 10.1006/dbio.2002.0852

[49] Flanagan-Steet H, Fox MA, Meyer D, Sanes JR (2005) Neuromuscular synapses can form in vivo by incorporation of initially aneural postsynaptic specializations. Development 132: 4471–4481. 16162647 10.1242/dev.02044

[50] Zeller J, Granato M (1999) The zebrafish diwanka gene controls an early step of motor growth cone migration. Development 126: 3461–3472. 10393124 10.1242/dev.126.15.3461

[51] Knapik EW, Goodman A, Atkinson OS, Roberts CT, Shiozawa M, et al (1996) A reference cross DNA panel for zebrafish (Danio rerio) anchored with simple sequence length polymorphisms. Development 123: 451–460. 9007262 10.1242/dev.123.1.451

