# Supplementary figures and images for: Plexin A3 and Turnout Regulate Motor Axonal Branch Morphogenesis in Zebrafish
Source: PLoS One. 2013 Jan 21;8(1):e54071. doi: 10.1371/journal.pone.0054071 (PMC3549987; doi:10.1371/journal.pone.0054071)

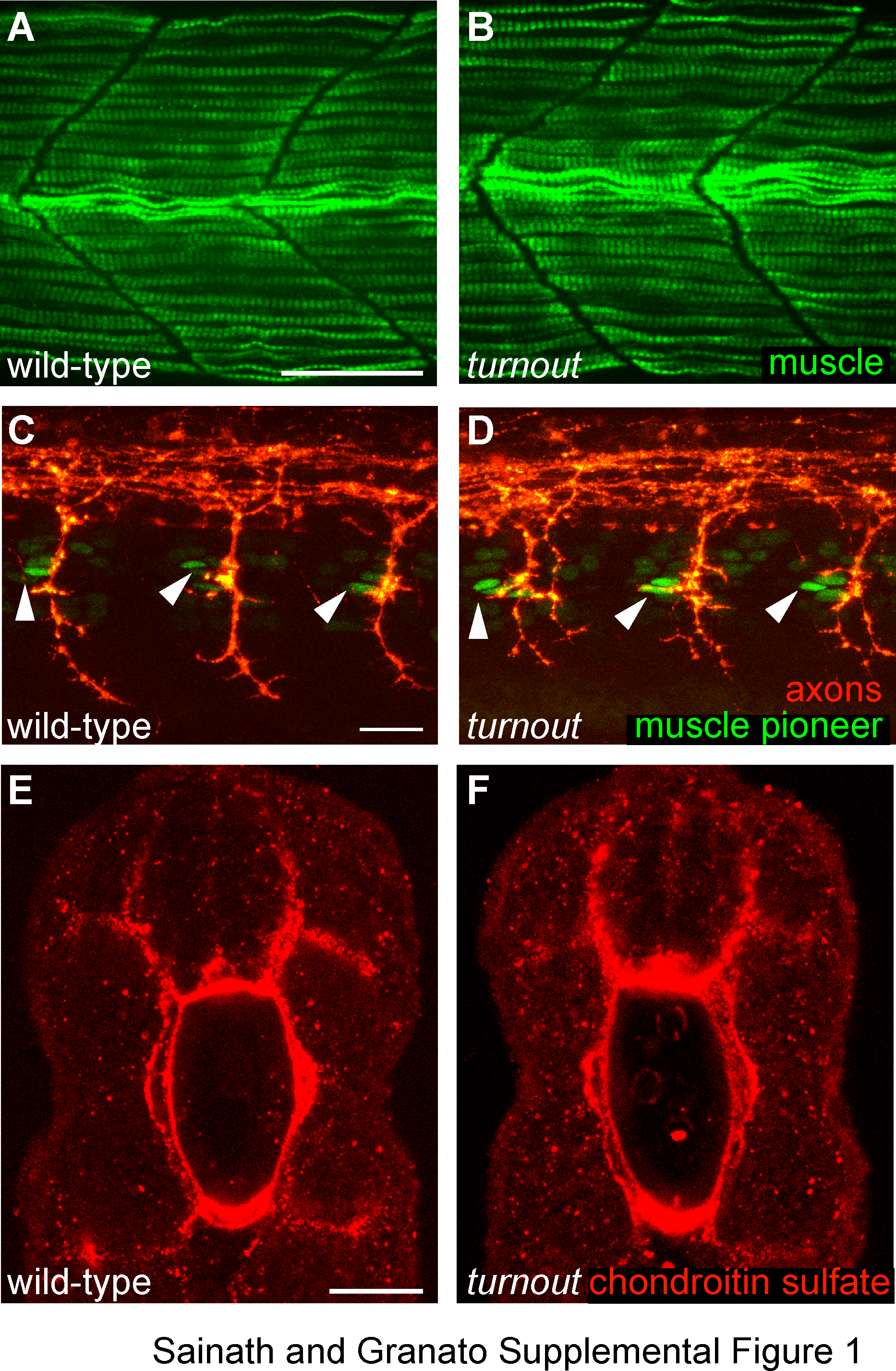

Supplement: Figure S1 — Muscle fiber development, muscle pioneer specification and position and extracellular matrix appear unaffected in turnout mutant embryos. (A, B) Lateral view of 28 hpf wild-type. Scale bar, 25 µm. (A) and turnout (B) mutant embryos stained with the F59 antibody which recognizes myosin heavy chain in adaxial cell derived slow-twitch muscles cells indicates appropriate muscle fiber development and somite morphology. (C, D) Lateral view of 25 hpf wild-type (C) and turnout (D) embryos stained with the 4D9 antibody, which recognizes the nuclear Engrailed epitope in muscle pioneers indicates the correct number and position of pioneers cells present in each hemisegment located rostrally at the horizontal myoseptum. Scale bar, 20 µm. (E, F) Cross section of 24 hpf wild-type (E) and turnout (F) embryos stained with the chondroitin sulfate proteoglycan antibody indicates proper deposition of the extracellular matrix surrounding the spinal cord and the notochord. Scale bar, 25 µm. (TIF) [file pone.0054071.s001.tif]
